# Sincronización entre la videodeglución y la electromiografía de superficie en pacientes con afectación neurológica y síntomas de disfagia

**DOI:** 10.7705/biomedica.6446

**Published:** 2022-12-01

**Authors:** Laura V. Suárez-Patiño, Andrés Orozco-Duque, Estefanía Pérez-Giraldo, Sebastián Roldán-Vasco, Juan Camilo Suárez-Escudero, Lillyana Martínez-Moreno

**Affiliations:** 1 Facultad de Ciencias Exactas y Aplicadas, Instituto Tecnológico Metropolitano, Medellín, Colombia Instituto Tecnológico Metropolitano Facultad de Ciencias Exactas y Aplicadas Instituto Tecnológico Metropolitano Medellín Colombia; 2 Facultad de Ingenierías, Instituto Tecnológico Metropolitano, Medellín, Colombia Instituto Tecnológico Metropolitano Facultad de Ingenierías Instituto Tecnológico Metropolitano Medellín Colombia; 3 Facultad de Ingeniería, Universidad de Antioquia, Medellín, Colombia Universidad de Antioquia Facultad de Ingeniería Universidad de Antioquia Medellín Colombia; 4 Facultad de Medicina, Escuela de Ciencias de la Salud, Universidad Pontificia Bolivariana, Medellín, Colombia Universidad Pontificia Bolivariana Facultad de Medicina Escuela de Ciencias de la Salud Universidad Pontificia Bolivariana Medellín Colombia; 5 Rehabilitación Integral, OFA IPS, Organización Fonoaudiológica E.U., Medellín, Colombia Rehabilitación Integral OFA IPS Organización Fonoaudiológica E.U. Medellín Colombia

**Keywords:** trastornos de deglución, manifestaciones neurológicas, procesamiento de señales asistido por computador, electromiografía, esclerosis múltiple, enfermedad de Parkinson, deglutition disorders, neurologic manifestations, signal processing, computer-assisted, electromyography, multiple sclerosis, Parkinson’s disease.

## Abstract

**Introducción.:**

La disfagia se define como la dificultad para movilizar la comida desde la boca hasta el estómago. La prueba diagnóstica para esta condición es la videofluoroscopia, la cual no es totalmente inocua pues utiliza radiación ionizante. La electromiografía de superficie registra la actividad eléctrica de los músculos de manera no invasiva, por lo que puede considerarse como una alternativa para evaluar la deglución y estudiar la disfagia.

**Objetivo.:**

Evaluar la relación entre los tiempos relativos de activación de los músculos implicados en la fase oral y faríngea de la deglución, con los movimientos registrados durante la videofluoroscopia.

**Materiales y métodos.:**

Se analizaron las señales de la electromiografía de superficie de 10 pacientes neurológicos con síntomas de disfagia, captadas en forma simultánea con la videofluoroscopia. Se suministraron 5 ml de yogur y 10 ml de agua, y 3 g de galleta. Se estudiaron bilateralmente los grupos musculares maseteros, suprahioideos e infrahioideos. Se analizó el paso del bolo por la línea mandibular, las valleculas y el músculo cricofaríngeo, correlacionándolo con el tiempo inicial y el final de la activación de cada uno de los grupos musculares.

**Resultados.:**

El tiempo promedio de la fase faríngea fue de 0,89 ± 0,12 s. En la mayoría de los casos, hubo activación muscular antes del paso por la línea mandibular y las valleculas. La terminación de la actividad muscular parece corresponder al momento en que se completa el paso del bolo alimenticio por el músculo cricofaríngeo.

**Conclusión.:**

Se determinaron los tiempos de actividad muscular, la duración de la fase faríngea y la secuencia de la activación de los grupos musculares involucrados en la deglución, mediante electromiografía de superficie, validada con la videofluoroscopia.

## Abstract (en)

**Introduction:**

Dysphagia is defined as the difficulty in transporting food and liquids from the mouth to the stomach. The gold standard to diagnose this condition is the videofluoroscopic swallowing study. However, it exposes patients to ionizing radiation. Surface electromyography is a non-radioactive alternative for dysphagia evaluation that records muscle electrical activity during swallowing.

**Objective:**

To evaluate the relationship between the relative activation times of the muscles involved in the oral and pharyngeal phases of swallowing and the kinematic events detected in the videofluoroscopy.

**Materials and methods:**

Electromiographic signals from ten patients with neurological involvement who presented symptoms of dysphagia were analyzed simultaneously with videofluoroscopy. Patients were given 5 ml of yogurt, 10 ml of water, and 3 g of crackers. Masseter, suprahyoid, and infrahyoid muscle groups were studied bilaterally. The bolus transit through the mandibular line, vallecula, and the cricopharyngeus muscle was analyzed in relation to the onset and offset times of each muscle group activation.

**Results:**

The average time of the pharyngeal phase was 0.89 ± 0.12 s. Muscle activation was mostly observed prior to the bolus transit through the mandibular line and vallecula. The end of the muscle activity suggested that the passage of the bolus through the cricopharyngeus muscle was almost complete.

**Conclusión:**

The muscle activity times, duration of the pharyngeal phase, and sequence of the muscle groups involved in swallowing were determined using sEMG validated with the videofluoroscopic swallowing study.

La deglución es un proceso fisiológico muy bien coordinado, que involucra varias conexiones a nivel neurológico y muscular para un eficiente y seguro transporte de líquidos y alimentos desde la boca hasta el estómago ^1^. La deglución consta de cuatro etapas: preparatoria oral, propulsiva oral, faríngea y esofágica. Cada etapa es regulada por diferentes centros neurológicos y funciones neuromusculares que reconfiguran la faringe de forma precisa, para poder cumplir con el acto de tragar ^2^^,^^3^.

La disfagia, o trastorno de la deglución, es la dificultad para movilizar de manera segura y eficaz el bolo alimenticio desde la cavidad oral hasta el estómago ^1^^,^^4^. Es de alta prevalencia, se presenta a cualquier edad y lo puede hacer de manera aislada o acompañando un amplio espectro de enfermedades. La disfagia puede afectar hasta el 12 % de la población general ^5^, llegando a tener una prevalencia de hasta el 33 % en adultos mayores ^6^^,^^7^. Según la localización fisiológica del trastorno, se clasifica en orofaríngea o esofágica. Además, se puede clasificar en disfagia de origen estructural, motora, iatrogénica o funcional, de acuerdo con su etiología ^8^.

La incidencia mundial de la disfagia funcional de origen neurológico se encuentra entre 400.000 y 800.000 casos al año ^9^. Por esta razón, la disfagia -en especial la orofaríngea-, se asocia frecuentemente a trastornos neurológicos como accidentes cerebrovasculares, enfermedades neurodegenerativas como la enfermedad de Parkinson, y procesos autoinmunitarios como la esclerosis múltiple; a su vez, estas condiciones son más prevalentes en la población de adultos mayores ^10^.

La disfagia produce diversos síntomas y signos que permiten detectarla, y establecer el déficit fisiológico asociado y su gravedad, mediante métodos de tamización, evaluaciones clínicas de la deglución y métodos instrumentales ^8^.

La disfagia puede detectarse mediante pruebas clínicas o métodos instrumentales ^11^. Los métodos instrumentales más empleados son la videofluoroscopia (*VideoFluoroscopic Swallowing Study*, VFSS) y la endoscopia (*Fiber Endoscopic Evaluation of Swallowing*, FEES) ^12^, las cuales permiten detectar alguna aspiración o penetración o alteraciones funcionales de la región implicada ^9^. El método diagnóstico de referencia para esta condición es la videofluoroscopia de la deglución ^13^; sin embargo, esta técnica no es inocua pues expone al paciente a radiación ionizante ^14^.

Lo anterior es suficiente razón para explorar otras alternativas no invasivas, como la electromiografía de superficie, técnica diseñada para registrar la actividad eléctrica de los músculos mediante electrodos superficiales ^15^^-^^17^. La electromiografía de superficie se ha utilizado para evaluar la disfagia ^18^ y detectar patrones de actividad muscular; además, diversos autores la han utilizado para evaluar la fisiología de la deglución (Chou W, Ou CZ, Lin BS, Ko MJ, Hu SP, Ting YM, *et al*. Wireless and wearable monitoring device for dysphagia evaluation. In: 2015 IEEE MTT-S International Microwave Workshop Series on RF and Wireless Technologies for Biomedical and Healthcare Applications, IMWS-BIO 2015 - Proceedings. Taiwan: IEEE MTT; 2015. p. 174-7.), tanto para la rehabilitación ^15^, el diagnóstico ^19^^,^^20^ duration from the maximum amplitude to the end of the swallowing activity (duration B y el análisis de la coordinación de la fase oral de la deglución ^21^^,^^22^. De esta manera, la electromiografía de superficie ofrece una alternativa no invasiva y de fácil acceso que eliminaría el impacto de los riesgos asociados con la radiación ionizante ^11^.

A pesar de que varias investigaciones ^19^^,^^21^^,^^23^ (Chou W, Ou CZ, Lin BS, Ko MJ, Hu SP, Ting YM, *et al*. Wireless and wearable monitoring device for dysphagia evaluation. In: 2015 IEEE MTT-S International Microwave Workshop Series on RF and Wireless Technologies for Biomedical and Healthcare Applications, IMWS-BIO 2015 - Proceedings. Taiwan: IEEE MTT; 2015. p. 174-7); Saijo R, Saotome K, Jayatilake D, Suzuki K. EMG signals based modelling of the initial phase of the swallowing process. 8th International IEEE/EMBS Conference on Neural Engineering, NER 2017, Shanghai, China, May 25-28, 2017. IEEE, 2017. 2017;78-81), revelan el potencial de esta técnica para el estudio de patrones deglutorios, la electromiografía de superficie no se utiliza aún ni para evaluar la deglución ni para diagnosticar la disfagia en entornos clínicos, ya que con ella solo se pueden describir indirectamente los procesos musculares involucrados en la deglución, dado que es limitado el seguimiento de la actividad muscular regional y no es posible aislar músculos u otras regiones para evaluarlos ^24^. Esto hace necesario validar la técnica mediante pruebas de referencia, con el fin de estandarizar su uso en la práctica clínica ^17^.

La carencia de estudios que validen este tipo de electromiografía, en combinación con pruebas instrumentales de referencia para evaluar la deglución, motiva el presente estudio preliminar sobre la sincronización entre las imágenes de la videofluoroscopia y las señales de la electromiografía de superficie, en pacientes neurológicos con síntomas de disfagia, puesto que permite evaluar los tiempos de activación de los músculos implicados en la fase oral y la faríngea de la deglución, con respecto a los movimientos detectados en la videofluoroscopia.

## Materiales y métodos

**Figura 1:**
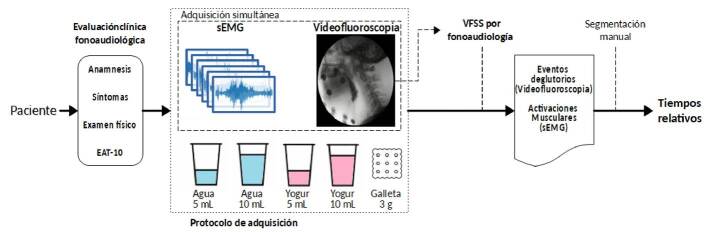
Esquema metodológico del estudio

El presente estudio, observacional y de corte transversal, incluyó pacientes con afectación neurológica que presentaban síntomas de disfagia, a los cuales se les practicó una videofluoroscopia simultáneamente con una electromiografía de varios grupos musculares. En el esquema metodológico empleado, una vez seleccionado el paciente, se hizo la evaluación fonoaudiológica con un examen clínico centrado en la deglución, seguido de la utilización del instrumento EAT-10 (*Eating Assessment Tool*) (figura 1). Posteriormente, se siguió un protocolo para obtener al mismo tiempo los datos de ambos estudios y evaluar la deglución (figura 1).

### Reclutamiento de pacientes

Se seleccionaron 10 pacientes adultos que hacían parte de un estudio de cohorte sobre la disfagia orofaríngea funcional de causas neurológicas y neuromusculares. Para ser seleccionado, el paciente debía tener diagnóstico de enfermedad neurológica central (enfermedad de Parkinson o esclerosis múltiple con patrón clínico de recaída y remisión) y síntomas de disfagia, con un puntaje de tres o más en el EAT-10. Se tomó como criterio de exclusión, el haber sido sometido a videofluoroscopia durante el año inmediatamente anterior a este estudio, o tener enfermedades del sistema nervioso periférico o enfermedades neuromusculares.

### Examen clínico

Antes de la electromiografía, una fonoaudióloga con experiencia y entrenamiento en deglución y disfagia hizo la evaluación clínica y se utilizó el EAT-10 validado en español para Colombia ^25^.

El EAT-10 es un instrumento analógico verbal, unidimensional, simple y de utilización breve, autoadministrado y de puntuación directa, que permite evaluar síntomas específicos de disfagia y el establecimiento inicial de la gravedad del síntoma ^26^. Posee un puntaje que va desde cero (“ningún problema”) hasta 40 (“mayor dificultad o percepción de la disfagia”). Se tomó como criterio de inclusión un puntaje del EAT-10 de 3 o más, debido a que es el punto de corte con mejor equilibrio entre sensibilidad y especificidad para la disfagia orofaríngea ^27^.

Los datos se obtuvieron en la IPS Hernán Ocazionez (Medellín, Colombia). De cada paciente, se obtuvo el consentimiento informado aprobado por el Comité de Ética de la Universidad Pontificia Bolivariana, sede Medellín, reunido el 14 de septiembre del 2020 (acta N°19 de 2020).

### Adquisición de señales e imágenes

**Figura 2:**
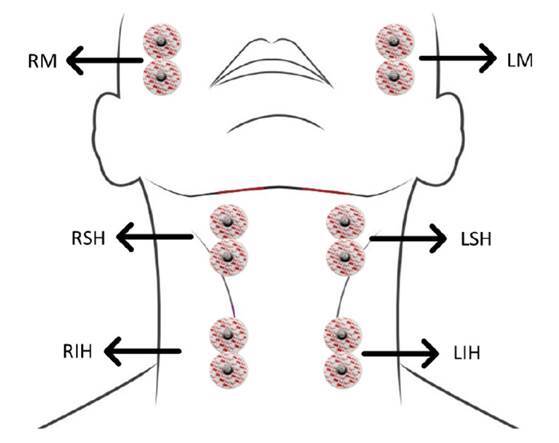
Posicionamiento de los electrodos superficiales sobre los grupos musculares de interés

Se registraron las señales bilaterales en los músculos maseteros (derecho e izquierdo: RM y LM), suprahioideos (derecho e izquierdo: RSH y LSH) e infrahioideos (derecho e izquierdo: RIH y LIH) (figura 2).

La activación de los últimos grupos musculares observada en la electromiografía es clave en la elevación mecánica superior y el desplazamiento anterior de la laringe ^28^, parámetro que se utiliza a la hora de interpretar la videofluoroscopia. Para la adquisición de las señales eléctricas, se utilizaron electrodos de superficie de referencia Ag/AgCl (3M® 2228) de 30 x 35 mm, 15 mm de diámetro en el área de gel y distancia de 25 mm entre electrodos.

La videofluoroscopia se practicó con el electromiógrafo Ultium™ EMG (Noraxon, USA), el cual se sincronizó con un arco en C (OEC Fluorostar, GE Healthcare) mediante conexión por puerto DVI (*Digital Visual Interface*). Cada paciente ingirió 5 ml de un líquido espeso (yogur) y 10 ml de un líquido claro (agua), así como 3 g de sólido seco (galleta). El protocolo no incluyó la alimentación a libre demanda por parte del paciente. Se usó sulfato de bario como medio de contraste (E-Z-HDTM). La deglución se evaluó en vista lateral, evitando la superposición visual de los electrodos de la electromiografía con las de estructuras anatómicas implicadas en la deglución.

Se aplicó un filtro pasabandas a cada una de las señales electromiográficas, con frecuencias de corte entre 90 y 250 Hz. Estas frecuencias se eligieron teniendo en cuenta lo descrito por Restrepo, *et al*., en el 2017 ^29^.

### Segmentación de señales

Con ayuda de fonoaudiólogos entrenados en deglución y disfagia, se validó la calidad de cada uno de los videos obtenidos y se determinaron los tiempos en el que el bolo alimenticio inicia la trayectoria en la boca, desciende por la faringe y llega a la porción proximal del esófago. Las imágenes de la videofluoroscopia se utilizaron para detectar tres momentos deglutorios: paso por la línea mandibular, paso por las valleculas epiglóticas y paso por el músculo cricofaríngeo o complejo del esfínter esofágico superior.

**Figura 3:**
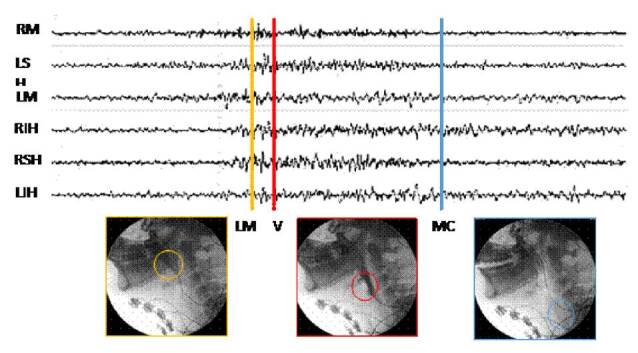
Posicionamiento de los marcadores sobre línea mandibular (LM), vallecula epiglótica (V) y músculo cricofaríngeo (MC)

Se establecieron marcadores de los tiempos en que sucede cada uno de estos eventos, con el propósito de validar si el paso del alimento por los puntos marcados corresponde a una activación muscular. Bajo visión videofluoroscópica, se posicionaron tres marcadores de eventos sobre una señal electromiográfica multicanal de los seis diferentes grupos musculares evaluados (figura 3).

Se identificó el tiempo en el cual el alimento pasó por la línea mandibular, las valleculas y el músculo cricofaríngeo; y se reconoció el momento en que la señales electromiográficas mostraron inicio y fin de la actividad muscular (*onset* y *offset*, respectivamente). Se calcularon las diferencias de tiempo asociadas con: *onset* y paso por la línea mandibular, *onset* y paso por las valleculas, *offset* y paso por la línea mandíbula, y *offset* y paso por el músculo cricofaríngeo. Además, se calculó la duración del segmento de activación de la señal electromiográfica y la duración de la fase faríngea. Finalmente, se calculó la media de dichos datos con su respectiva desviación estándar, con el fin de evaluar la relación entre los registros de las señales eléctricas y los de los momentos fisiológicos en la videofluoroscopia. El paso del alimento por la línea mandibular se estableció como punto de referencia.

Se utilizó la prueba U de Mann-Whitney U para comparar los diferentes tiempos relativos con la duración de la actividad muscular y la de la fase faríngea, y relacionarlos con las diferentes consistencias y volúmenes del bolo alimenticio.

## Resultados

### Descripción clínica

Las características clínicas de los pacientes se resumen en el cuadro 1. Este grupo de pacientes se encuentra pareado por sexo (5 hombres y 5 mujeres), con una edad promedio de 58 ± 8 años. Cada uno de los pacientes recibió alimentación por vía oral en el momento de la prueba.

**Cuadro 1:** Características clínicas de los pacientes

**Código**	**Sexo**	**Edad**	**Diagnóstico**	Años de evolución del diagnóstico	**Comorbilidades**	Tolerancia a líquidos claros y espesos*	Tolerancia a sólidos secos*	Sitio sensación de atascamiento comida*	Puntaje total EAT- 10**
01	F	52	EM	6	Reflujo gastroesofágico, disautonomía	Sí	No	Cuello	12
02	F	56	EM	19	Hipotiroidismo, linfedema de miembros inferiores, reflujo gastroesofágico, bypass gástrico hace 12 años	Sí	No	No	12
03	F	50	EM	17	Marcha alterada, fasciculaciones linguales	Sí	No	Cuello	22
04	M	42	EP	6	Disartria	Sí	Sí	Cuello	18
05	M	61	EP	10	Reflujo gastroesofágico, disfonía	Sí	No	Cuello	19
06	M	64	EP	4	Apnea obstructiva del sueño, disautonomía, fonastenia	Sí	No	Tórax	23
07	F	67	EP	2	Hipertensión arterial, dislipidemia, hipotiroidismo, gastritis crónica	Sí	No	Boca	15
08	M	64	EP	3	Trastorno depresivo y ansiedad, infecciones urinarias a repetición, marcha alterada, disartria	Sí	No	Boca	26
09	M	66	EP	4	Trastorno de ansiedad, disartria, insuficiencia cardíaca, fibrilación auricular, bypass coronario más cuatro stent, síndrome de apnea obstructiva del sueño, hipertensión arterial, hernia hiatal operada hace 10 años, epiglotitis con drenaje quirúrgico, Marcha alterada, disartria y fasciculaciones linguales	Sí	No	No	12
10	F	52	EM	7	Sí	Sí	No	No	11

F: femenino; M: masculino; EM: esclerosis múltiple; EP: enfermedad de Parkinson

* Dato reportado por el paciente

** EAT-10: *Eating Assessment* Tool-10

Ningún paciente recibía oxígeno suplementario, ni tenía gastrostomía ni colostomía durante la prueba. Ninguno se encontraba en rehabilitación mediante terapia respiratoria o terapia para la deglución, en el momento inmediatamente anterior a la práctica de la prueba. Un paciente (código 09) se encontraba activo en terapia física. Ninguno de los pacientes había sido sometido a intubación durante más de una semana o a traqueostomía en los últimos seis meses, ni tampoco había sufrido neumonía por aspiración.

Todos los pacientes fueron evaluados mediante videofluoroscopia, cumpliendo el protocolo establecido (cuadro 2).

**Cuadro 2:** Resultados de la videodeglución en cada paciente

Código	Resultado VFSS
01	Alteración en función faríngea con episodios de acumulación en estructuras faríngeas con consistencias espesas y sólidas. Sin episodios de penetración, broncoaspiración ni aspiraciones silentes
02	Sin alteración en biomecánica deglutoria con consistencia espesa, líquida o sólida. Adecuada eficacia y eficiencia deglutorias. Sin episodios de penetración, broncoaspiración ni aspiraciones silentes
03	Alteración en función faríngea con episodios de acumulación en estructuras faríngeas con consistencias espesas y sólidas. Sin episodios de penetración, broncoaspiración ni aspiraciones silentes
04	Alteración en función faríngea, disfagia de tipo neurogénica. Sin episodios de penetración, broncoaspiración ni aspiraciones silentes.
05	Alteración en función faríngea con episodios de acumulación en estructuras faríngeas con consistencias espesas y sólidas. Sin episodios de penetración, broncoaspiración ni aspiraciones silentes
06	Sin alteración en biomecánica deglutoria con consistencia espesa, líquida ni sólida. Adecuada eficacia y eficiencia deglutoria. Sin episodios de penetración, broncoaspiración ni aspiraciones silentes
07	Función oral de la deglución con proceso oromotor lento, falta de fuerza y movilidad en la ejecución de *praxias oromotoras*. Presenta derrame anterior y degluciones fraccionadas. Aumento de latencia con todas las consistencias. Sin alteración en función faríngea de la deglución. Adecuada eficacia y eficiencia deglutoria. Sin episodios de penetración, broncoaspiración ni aspiraciones silentes.
08	Alteración en función oral de la deglución con pobre control de saliva en cavidad oral y proceso oromotor con falta de fuerza, movilidad y coordinación. Alteración en función faríngea con episodios de acumulación en estructuras faríngeas, con consistencias espesas y sólidas. Sin episodios de penetración, broncoaspiración ni aspiraciones silentes.
09	Alteración en función faríngea, con episodios de acumulación en estructuras faríngeas con consistencias espesas. Adecuada funcionalidad faríngea, con consistencias líquidas y sólidas. Sin episodios de penetración, broncoaspiración ni aspiraciones silentes.
10	Alteración en función faríngea con episodios de acumulación en estructuras faríngeas con consistencias espesas. Adecuada funcionalidad faríngea con consistencias líquidas y sólidas. Sin episodios de penetración, broncoaspiración ni aspiraciones silentes.

Hubo seis pacientes con enfermedad de Parkinson, con dos o más años de evolución, y cuatro pacientes con esclerosis múltiple, con seis o más años de evolución (cuadro 1).

El promedio del puntaje del EAT-10 de todos los pacientes en general, fue de 17 puntos, mientras que, en aquellos con enfermedad de Parkinson, fue de 18,83, y en aquellos con esclerosis múltiple, fue de 14,25. El puntaje más alto de autopercepción de síntomas se obtuvo en: los pacientes con código 03 (diagnóstico de esclerosis múltiple con fasciculaciones linguales y compromiso de la función faríngea en la videofluoroscopia con alimentos de consistencia espesa o sólida); en aquellos con código 06 (diagnóstico de enfermedad de Parkinson con fonastenia y sin alteración de la deglución en la videofluoroscopia, sin importar la consistencia del alimento) y, finalmente, pacientes con código 08 (diagnóstico de enfermedad de Parkinson con disartria y alteración de la función oral y faríngea durante la deglución en la videofluoroscopia). Se debe tener presente que los síntomas de disfagia no confirman una alteración de la deglución.

Mediante la videofluoroscopia, se confirmó que la deglución era normal en los pacientes con código 02 (esclerosis múltiple) o código 06 (enfermedad de Parkinson), y estaba alterada por compromiso de la función faríngea, en aquellos con códigos 01, 03, 04, 05, 09 o 10. Además, hubo alteración de la deglución en un paciente con código 07 por compromiso de la función oral y, en otro con código 08, por compromiso de la función oral y faríngea.

En general, la disfagia en los pacientes del presente estudio no era grave, pues la vía de alimentación era oral, sin gastrostomía u otras vías alternas de alimentación; además, en ninguno se observaron episodios de penetración o broncoaspiración, ni aspiraciones silentes.

### Análisis de señales

A continuación, se presentan los resultados de los tiempos relativos promedio entre *onset* y *offset* de las activaciones musculares y los eventos deglutorios observados por videofluoroscopia. Dichos resultados se basan en los promedios de todos los pacientes; en general, se observó una gran variabilidad.

A partir de la medición de los tiempos relativos, se observó que, en la mayoría de los grupos musculares y las tareas deglutorias, ocurrió una activación previa con respecto al punto de referencia (paso por la línea mandibular). Esto se evidencia con el promedio de tiempo negativo entre el *onset* de la activación muscular y el paso por la línea mandibular (cuadros 3, 4 y 5).

**Cuadro 3:** Duración en segundos del tiempo transcurrido entre los puntos marcados para cada uno de los grupos musculares para la deglución de yogur

	Grupo muscular	*Onset- Paso* LM	***Onset- Paso* V**	*Offset -Paso* LM	*Offset- Paso* MC	Duración sEMG	Duración fase faríngea por VFSS
Yogur	RM	-0,26 ± 0,31	-0,45 ± 0,24	0,61 ± 0,41	-0,25 ± 0,32	0,87 ± 0,44	0,87 ± 0,25
5 ml	LM	-0,41 ± 0,74	-0,60 ± 0,70	0,38 ± 0,64	-0,49 ± 0,55	0,79 ± 0,49	
	RSH	-0,03 ± 0,44	-0,22 ± 0,44	0,96 ± 0,87	0,10 ± 0,85	0,99 ± 0,47	
	LSH	0,04 ± 0,61	-0,15 ± 0,60	1,04 ± 0,70	0,17 ± 0,67	1,00 ± 0,30	
	RIH	-0,10 ± 0,41	-0,29 ± 0,32	1,19 ± 0,94	0,32 ± 0,85	1,29 ± 1,02	
	LIH	-0,10 ± 0,38	-0,31 ± 0,30	1,25 ± 0,90	0,39 ± 1,04	1,38 ± 0,89	
Yogur	RM	-0,81 ± 0,88	-0,91 ± 0,93	0,01 ± 1,00	-0,82 ± 1,04	0,81 ± 0,36	0,83 ± 0,19
10 ml	LM	-0,72 ± 0,83	-0,83 ± 0,87	-0,07 ± 1,02	-0,90 ± 1,02	0,65 ± 0,41	
	RSH	-0,38 ± 0,84	-0,48 ± 0,84	0,72 ± 0,39	-0,11 ± 0,39	1,09 ± 1,12	
	LSH	-0,41 ± 0,85	-0,51 ± 0,86	0,85 ± 0,53	0,02 ± 0,55	1,26 ± 1,32	
	RIH	-0,31 ± 0,88	-0,42 ± 0,88	0,76 ± 0,45	-0,07 ± 0,41	1,07 ± 1,17	
	LIH	-0,31 ± 0,91	-0,41 ± 0,91	0,79 ± 0,45	-0,03 ± 0,41	1,10 ± 1,20	

VFSS: *Videofluoroscopic Swallowing Study*

Paso LM: paso por línea mandibular; paso V: paso por valléculas: paso MC: paso por músculo cricofaríngeo; sEMG: electromiografía de superficie; VFSS: videofluoroscopia; RM: masetero derecho; LM: masetero izquierdo; RSH: suprahiodeo derecho; LSH: suprahiodeo izquierdo; RIH: infrahiodeo derecho; LIH: infrahiodeo izquierdo

**Cuadro 4:** Duración del tiempo medido en segundos entre los puntos marcados para cada uno de los grupos musculares para la deglución de agua

	Grupo muscular	*Onset- Paso* LM	*Onset- Paso* V	*Offset -Paso* LM	*Offset- Paso* MC	Duración EMG	Duración fase faríngea por VFSS
Agua	RM	-0,45 ± 0,98	-0,60 ± 0,99	0,68 ± 1,12	-0,14 ± 1,14	1,13 ± 0,77	0,82 ± 0,14
5 ml	LM	-0,76 ± 0,83	-0,90 ± 0,81	0,39 ± 0,47	-0,44 ± 0,49	1,14 ± 0,93	
	RSH	-0,18 ± 0,64	-0,32 ± 0,66	0,74 ± 0,47	-0,08 ± 0,49	0,92 ± 0,42	
	LSH	-0,18 ± 0,89	-0,32 ± 0,91	0,82 ± 0,42	-0,01 ± 0,48	0,99 ± 0,75	
	RIH	-0,23 ± 0,56	-0,37 ± 0,55	0,75 ± 0,36	-0,07 ± 0,40	0,98 ± 0,41	
	LIH	-0,33 ± 0,58	-0,47 ± 0,56	0,87 ± 0,57	0,04 ± 0,56	1,19 ± 0,80	
Agua	RM	-0,72 ± 0,79	-0,82 ± 0,80	0,54 ± 0,46	-0,27 ± 0,55	1,26 ± 1,13	0,81 ± 0,28
10 ml	LM	-0,80 ± 0,81	-0,91 ± 0,82	0,47 ± 0,47	-0,34 ± 0,53	1,27 ± 1,16	
	RSH	-0,37 ± 0,90	-0,48 ± 0,91	1,06 ± 0,70	0,25 ± 0,81	1,43 ± 1,37	
	LSH	-0,43 ± 0,92	-0,53 ± 0,94	1,24 ± 0,75	0,43 ± 0,63	1,37 ± 1,34	
	RIH	-0,55 ± 0,90	-0,65 ± 0,91	0,85 ± 0,63	0,04 ± 0,72	1,41 ± 1,18	
	LIH	-0,37 ± 0,90	-0,47 ± 0,92	1,11 ± 0,57	0,29 ± 0,52	1,47 ± 1,13	

VFSS: *Videofluoroscopic Swallowing Study*

Paso LM: paso por línea mandibular; Ppaso V: paso por valléculas: paso MC: paso por músculo cricofaríngeo; sEMG: electromiografía de superficie; VFSS: videofluoroscopia; RM: masetero derecho; LM: masetero izquierdo; RSH: suprahiodeo derecho; LSH: suprahiodeo izquierdo; RIH: infrahiodeo derecho; LIH: infrahiodeo izquierdo

**Cuadro 5:** Duración del tiempo medido en segundos entre los puntos marcados para cada uno de los grupos musculares para la deglución de galleta

	Grupo muscular	*Onset-Paso* LM	*Onset- Paso* V	*Offset -Paso* LM	*Offset- Paso* MC	Duración EMG	Duración fase faríngea por VFSS
Galleta	RM	-0,63 ± 0,58	-0,86 ± 0,51	0,65 ± 0,49	-0,47 ± 0,27	1,27 ± 0,37	1,12 ± 0,46
	LM	-0,70 ± 0,72	-0,94 ± 0,65	0,63 ± 0,55	-0,48 ± 0,46	1,33 ± 0,65	
	RSH	-0,51 ± 0,54	-0,75 ± 0,56	0,93 ± 0,54	-0,19 ± 0,52	1,44 ± 0,83	
	LSH	-0,44 ± 0,48	-0,68 ± 0,50	0,92 ± 0,60	-0,20 ± 0,63	1,36 ± 0,77	
	RIH	-0,10 ± 0,38	-0,33 ± 0,21	1,37 ± 1,39	0,25 ± 1,18	1,47 ± 1,15	
	LIH	-0,09 ± 0,37	-0,33 ± 0,21	1,47 ± 1,78	0,35 ± 1,61	1,56 ± 1,52	

VFSS: *Videofluoroscopic Swallowing Study*

Paso LM: paso por línea mandibular; paso V: paso por valléculas: paso MC: paso por músculo cricofaríngeo; sEMG: electromiografía de superficie; VFSS: videofluoroscopia; RM: masetero derecho; LM: masetero izquierdo; RSH: suprahiodeo derecho; LSH: suprahiodeo izquierdo; RIH: infrahiodeo derecho; LIH: infrahiodeo izquierdo

Los resultados muestran la bilateralidad del proceso, dado que los tiempos de activación de los pares de grupos musculares tienden a ser similares. Mediante la prueba U de Mann-Whitney, se observa que no hay diferencias estadísticamente significativas entre los tiempos de activación de los músculos maseteros (p=0,18), suprahioideos (p=0,47) o infrahioideos (p=0,4), al comparar el lado derecho con el izquierdo.

Para la mayoría de las tareas deglutorias, con excepción de los 5 ml de líquido claro y de líquido espeso, el proceso se inicia por activación de los músculos maseteros, seguidos de los suprahioideos y, finalmente, de los infrahioideos. Con los bolos líquidos de 5 ml, no fue tan clara la activación preliminar de los suprahioideos frente a los infrahioideos (cuadros 3 y 4).

Para los diferentes grupos musculares, existe activación previa al paso del alimento por la línea mandibular y por las valleculas, a excepción del suprahioideo izquierdo con 5 ml de líquido espeso, el cual se activó después del paso por la línea mandibular. Se observó que el fin de la actividad de los músculos infrahioideos está más cerca del paso por el músculo cricofaríngeo, que la de los suprahioideos y los maseteros (cuadros 3-5).

Durante la ingestión del sólido seco, el final de la actividad muscular de los infrahioideos sufrió un retardo en relación con la deglución de líquidos. Sin embargo, estos músculos se desactivaron después del paso del alimento por el punto del músculo cricofaríngeo (cuadro 4).

Por otra parte, la duración de la fase faríngea es más prolongada con los volúmenes de 5 ml que con los de 10 ml (cuadros 3 y 4).

**Figura 4:**
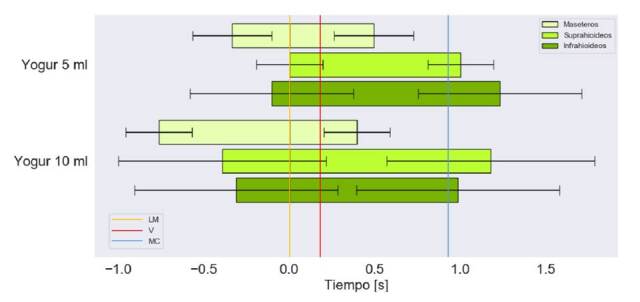
Tiempo de activación en segundos de los grupos musculares bilaterales para la deglución de yogur. Marcas en el tiempo del paso por la línea mandibular (LM), la de las valléculas (V) y la del músculo cricofaríngeo (MC)

**Figura 5:**
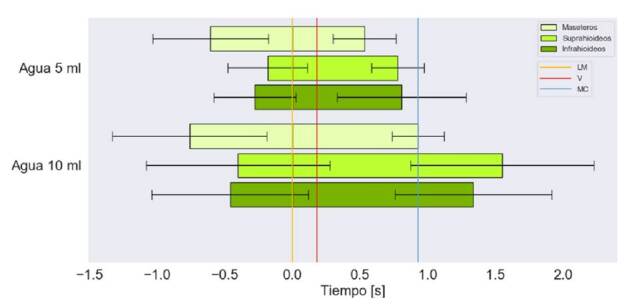
Tiempo de activación en segundos de los grupos musculares bilaterales para la deglución de agua. Marcas en el tiempo del paso por la línea mandibular (LM), la de las valleculas (V) y la del músculo cricofaríngeo (MC)

En las figuras 4, 5 y 6, se muestra el tiempo de activación de los grupos musculares bilaterales respecto a las diferentes consistencias y volúmenes, y, además, el registro del momento en el que el alimento pasa por la línea mandibular, las valleculas y el músculo cricofaríngeo.

**Figura 6:**
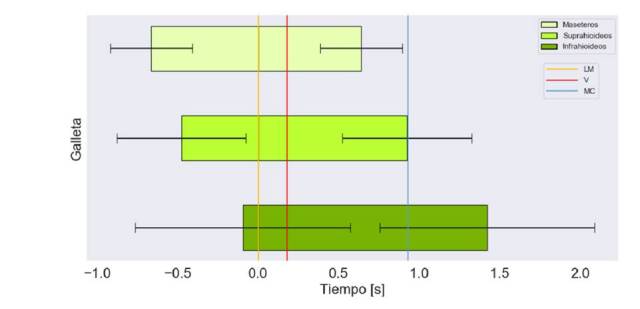
Tiempo de activación en segundos de los grupos musculares bilaterales para la deglución de galletas. Marcas en el tiempo del paso por paso por la línea mandibular (LM), la de las valleculas (V) y la del músculo cricofaríngeo (MC)

El tiempo de activación más prolongado se presentó con los 10 ml de líquido espeso. Se observó que, con ambos volúmenes, los grupos musculares tienden a iniciar su activación antes de que el alimento pase por la línea mandibular, aunque con 5 ml de líquido espeso, los suprahioideos se activan casi en forma simultánea con el paso del bolo por dicho punto (figura 4).

Con ambos volúmenes, se observa que los maseteros se encontraban inactivos antes del paso del alimento por el músculo cricofaríngeo; además, las desviaciones estándar más grandes se relacionaron con los músculos suprahioideos e infrahioideos, con la ingestión de 10 ml de líquido espeso.

Los maseteros se activan antes que los demás grupos musculares y, también, son los primeros en desactivarse. Con 10 ml de líquido espeso, los suprahioideos se activan antes que los infrahioideos y estos últimos se desactivan primero; lo contrario sucede con 5 ml.

Durante la ingestión de líquido claro, el orden de activación de los grupos musculares fue el siguiente: maseteros, infrahioideos y suprahioideos. Por otra parte, la desactivación tiende a ocurrir de forma diferente según el volumen: con 5 ml, tiende a desactivarse primero el grupo suprahioideo y, con 10 ml, tiende a hacerlo primero el grupo infrahioideo (figura 5).

Para los diferentes volúmenes de líquido claro, la activación muscular se inició antes del paso del alimento por la línea mandibular. En general, a mayor volumen, es mayor el tiempo de activación, independientemente de la consistencia del líquido (figuras 4 y 5). Con 5 ml, todos los músculos se desactivaron antes del paso del alimento por el punto del músculo cricofaríngeo (figura 5).

El orden de activación y de desactivación de los grupos musculares es el mismo: maseteros, suprahioideos e infrahioideos (figura 6). A diferencia de lo que observó con los suprahioideos y los maseteros, el *onset* de los infrahioideos estuvo cerca (0,1 ± 0,04 s) del momento en que el alimento pasaba por la línea mandibular. En promedio, el *offset* de los suprahioideos coincidió con el paso del alimento por el músculo cricofaríngeo.

Los maseteros se inactivaron antes del paso de la galleta por el músculo cricofaríngeo, comportamiento que fue similar al del líquido espeso y el claro (figura 6). Finalmente, la duración de la activación muscular de los infrahioideos mostró mayor variabilidad en comparación con lo que se muestra en las figuras 4 y 5*.*

## Discusión

En el presente estudio se expone de manera preliminar un patrón de activación de los músculos involucrados en las fases oral y faríngea de la deglución con registros electromiográficos, en relación con tres momentos observados bajo videofluoroscopia: paso del bolo alimenticio por la línea mandibular, por las valleculas y por el músculo cricofaríngeo. Según la revisión bibliográfica, este sería el primer trabajo que explora la sincronización entre la electromiografía de superficie y la videofluoroscopia en pacientes con compromiso neurológico y síntomas de disfagia.

La esclerosis múltiple en los pacientes de este estudio presentó un patrón de recaída y remisión y no su forma primaria progresiva, que es la que más compromiso deglutorio produce. Se ha informado que entre el 24 y el 34 % de los pacientes con esclerosis múltiple sufren disfagia, cifra que puede llegar al 65 % cuando hay afectación grave debido al proceso desmielinizante primario ^30^.

Entre las enfermedades neurodegenerativas, sobresale la de Parkinson, pues es una de las que más capacidad tiene de producir disfagia, tanto orofaríngea como esofágica, como consecuencia de la degeneración de las vías dopaminérgicas en la sustancia negra, el cuerpo estriado y el sistema nervioso entérico. Entre el 30 y el 82 % de los pacientes con enfermedad de Parkinson sufren disfagia ^31^, pero, cuando se evalúa la deglución mediante videofluoroscopia, se reportan anomalías en la fase orofaríngea en 75 a 97 % de los casos. ^32^. Según los resultados obtenidos durante el estudio, no se observó disfagia en las fases iniciales de esta enfermedad, lo que puede explicar la deglución normal vista en la videofluoroscopia del paciente con código 06 ^33^.

La etapa faríngea de la deglución, punto central de este estudio marcado entre la línea de la mandíbula y la del músculo cricofaríngeo, se caracteriza por el paso del bolo de la faringe al esófago proximal y la oclusión de la laringe para la protección de la vía respiratoria; a su vez, el hueso hioides se eleva, y el esfínter esofágico superior se relaja y se abre. Esta se considera la etapa más compleja de la deglución, con una duración aproximada de un segundo ^34^.

Una manera de establecer la congruencia de los datos obtenidos es analizando la duración de la fase faríngea mediante videofluoroscopia (cuadros 3-5); su duración fue de 0,89 ± 0,12 segundos.

No se encontraron diferencias estadísticamente significativas entre consistencias (p=0,14) ni entre volúmenes (p=0,35*)*. La duración de la fase faríngea y los límites de la desviación estándar encontrados en este estudio, corresponden con los reportados en la literatura científica. Lee, *et al.,* en el 2020, reportaron una respuesta del reflejo de deglución en la fase faríngea de 0,53 ± 0,64 s en personas de edad avanzada (mayores de 65 años) ^35^. De la misma manera, Park, *et al.,* en el 2017, obtuvieron un tiempo de tránsito faríngeo de 0,84 ± 0,12 s, para 5 ml de líquido claro; no obstante, estos resultados se obtuvieron en personas sanas con edad cercana a los 30 años ^36^.

En el presente estudio, al analizar las señales electromiográficas obtenidas con la ingestión de 5 ml de líquido claro o espeso, la activación de los músculos infrahioideos precede a la de los suprahioideos, lo que coincide con un estudio previo en casos y controles (figuras 4 y 5) ^37^. Con 10 ml de líquido espeso y con 3 g de sólido seco, la activación de los músculos suprahioideos precede a la de los infrahioideos. Esta contradicción frente a los demás volúmenes, también ha sido reportada previamente en personas sanas ^38^.

El tiempo de actividad más prolongado corresponde a los infrahioideos (1,29 ± 0,19 s), mientras que la actividad menos prolongada corresponde a los suprahioideos (1,18 ± 0,2 s); este hallazgo es similar a lo reportado por Koyama, *et al.*, en el 2021 (cuadros 3-5) ^37^.

Las pruebas estadísticas entre el inicio de la actividad muscular y el paso por la línea mandibular, no mostraron diferencias estadísticamente significativas: con volúmenes de 5 ml, entre maseteros (p=0,12), suprahioideos (p=0,11) e infrahioideos (p=0,11); con volúmenes de 10 ml, entre maseteros (p=0,5), suprahioideos (p=0,35) e infrahioideos (p=0,11); ni entre consistencias de líquido espeso y líquido claro, entre maseteros (p=0,28), suprahioideos (p=0,33) e infrahioideos (p=0,054); tampoco, entre bolos de líquidos espesos, entre maseteros (p=0,12), suprahioideos (p=0,12) e infrahioideos (p=0,11); ni entre bolos de líquidos claros, entre maseteros (p=0,35), suprahioideos (p=0,11) e infrahioideos (p=0,35).

En el 73,81 % de los casos, el inicio de la activación muscular de los maseteros, suprahioideos e infrahioideos antecede el paso del bolo por la línea mandibular, mientras que en el 93,81 %, antecede al paso por la vallecula. Esto coincide con lo informado por Park, *et al.* (2017), quienes demostraron, mediante electrodos de aguja, que existe una activación muscular previa al momento en que el bolo está pasando por la vallecula ^36^. Asimismo, el 65,24 % de los finales de activación antecede al paso del alimento por el músculo cricofaríngeo. Esto permite inferir un patrón electromiográfico de activación asociado con el tránsito del bolo en la fase faríngea, en la cual la activación muscular precede a los fenómenos mecánicos de la deglución.

Dado que este es el primer estudio en que se compara la electromiografía de superficie con la videofluoroscopia practicadas de manera sincrónica, estos valores no se pueden contrastar con los informados en la literatura científica, razón por la cual consideramos que es una contribución.

La amplia desviación estándar de los resultados puede deberse a la variabilidad en la fisiología de la deglución asociada con la edad, la enfermedad, el volumen y la consistencia del bolo alimenticio, tal y como lo reportan Poorjavad, *et al*. (2017), y Lee, *et al.* (2020) ^35^^,^^39^.

El presente trabajo muestra algunas limitaciones, especialmente en la composición de la base de datos, dado que el grupo de muestra es pequeño (n=10) y se exponen dos diferentes tipos de enfermedades neurológicas de origen central, la esclerosis múltiple y la enfermedad de Parkinson.

Asimismo, en este estudio se suministraron volúmenes de alimentos inferiores al del límite de disfagia, establecido en 20 ml, aproximadamente ^40^; además, el no tomar alimento según la demanda, puede ocasionar que el paciente no manifieste penetración u aspiración durante la prueba.

En este estudio, se presenta un análisis exploratorio en etapa de desarrollo, previo a una fase de validación. Aunque es un estudio preliminar, se dio a conocer el tiempo de actividad muscular, la duración de la fase faríngea y la secuencia de los grupos musculares involucrados en la deglución, mediante la electromiografía de superficie sincronizada con la videofluoroscopia. En los resultados obtenidos, se puede evidenciar la compleja dinámica neuromuscular de la deglución y la disfagia.

Como trabajo futuro, se espera ampliar la muestra de pacientes para aumentar la capacidad de generalizar los resultados obtenidos en el presente estudio. El principal aporte de este estudio es el hallazgo de correlaciones entre la información obtenida con la electromiografía y el tránsito del bolo observado mediante videofluoroscopia, lo cual permite generar información para la utilización de la electromiografía en la evaluación de la disfagia.

Esto permitiría desarrollar métodos no invasivos para el seguimiento de los pacientes, de tal manera que la videofluoroscopia como método estándar sea utilizado en el diagnóstico, pero complementada con la electromiografía de superficie para el seguimiento, minimizando la exposición a radiación ionizante en los pacientes. Por lo anterior, este estudio tiene un potencial impacto frente a la disponibilidad de métodos no invasivos que puedan ser llevados a la práctica clínica, y que contribuyan en el proceso de tamización, seguimiento y rehabilitación de las personas con síntomas de disfagia.
